# The association of measures of body shape and adiposity with incidence of cardiometabolic disease from an ageing perspective

**DOI:** 10.1007/s11357-022-00654-9

**Published:** 2022-09-21

**Authors:** Fleur L. Meulmeester, Ko Willems van Dijk, Simon P. Mooijaart, Diana van Heemst, Raymond Noordam

**Affiliations:** 1 Department of Internal Medicine, Section of Gerontology and Geriatrics, Leiden University Medical Center, PO Box 9600, 2300RC Leiden, The Netherlands; 2 Department of Human Genetics, Leiden University Medical Center, Leiden, The Netherlands; 3 Department of Internal Medicine, Division of Endocrinology, Leiden University Medical Center, Leiden, The Netherlands

**Keywords:** Adiposity, Body composition, Cardiometabolic diseases, Geroscience, UK Biobank

## Abstract

While obesity increases the risk of developing cardiometabolic diseases (CMDs), these associations seem to attenuate with increasing age, albeit studied poorly. The present study aimed to investigate the associations between adiposity and CMDs in sex-specific groups of chronological age and leukocyte telomere length (LTL) as a measure of biological age. We investigated the associations between BMI, a body shape index, waist-to-hip ratio (adjusted for BMI) and total body fat, and incident coronary artery disease (CAD), type 2 diabetes (T2D) and ischemic stroke (IS) in 413,017 European-ancestry participants of the UK Biobank without CMD at baseline. We assessed the change in the associations between adiposity and CMD over strata of increasing chronological age or decreasing LTL. Participants (56% women) had a median (IQR) age of 57.0 (50.0–63.0) years. The median follow-up time was 12 years. People with higher BMI had a higher risk of incident CAD (HR 1.14 (95% confidence interval [CI] 1.13, 1.16)), T2D (HR 1.70 (95% CI 1.68, 1.72)) and IS (HR 1.09 (95% CI 1.06, 1.12)). In groups based on chronological age and LTL, adiposity measures were associated with higher risk of CAD and T2D in both men and women, but these associations attenuated with increasing chronological age (*P*_interactions_ < 0.001), but not with decreasing LTL (*P*_interaction_ men = 0.85; *P*_interaction_ women = 0.27). Increased (abdominal) adiposity was associated with higher risk of incident CMDs, which attenuated with increasing chronological age but not with decreasing LTL. Future research may validate these findings using different measures of biological age.

**Supplementary Information:**

The online version contains supplementary material available at 10.1007/s11357-022-00654-9.

## Introduction

Obesity is one of the main risk factors for developing cardiometabolic diseases (CMDs) [1, 2]. The causal relationship between obesity and CMDs has been studied extensively [3–7], showing that obesity causes, among others, an increased risk of hypertension [5, 6], coronary heart disease (CHD) [3–5] and type 2 diabetes (T2D) [3, 4, 7]. Interestingly, recent Mendelian randomisation (MR) studies showed that increasing age attenuated the association between body mass index (BMI) and coronary artery disease (CAD) [8], and T2D [9].

However, these MR studies have some limitations given that only BMI was investigated as a measure of increased adiposity, and stratified based on chronological age, whereas it is known that heterogeneity in the ageing state of an individual increases with increasing chronological age [10]. For example, body shape and composition have been shown to change over the course of life [11]. Although the approximation of adiposity defined as BMI is a reasonably accurate predictor at the population level [12, 13], it is a rather unspecific measure of body shape and composition and may therefore not provide the complete picture of an individual’s adiposity, particularly at older age [14]. This emphasises the importance of investigating the associations of multiple, more adiposity-specific measurements of obesity besides BMI, while considering the (chronological) age-dependent effects on body shape and composition, to study the contribution of adiposity to the onset and development of CMDs in more detail.

Heterogeneity at an older age is also the result of the ageing process itself. Ageing is driven by certain key mechanisms—*the hallmarks of ageing* [15]. Although these mechanisms may occur in every ageing individual, the hallmarks of ageing do not necessarily develop at the same rate for every person. Accordingly, individuals have been identified with exceptional longevity [16] or “healthy” ageing, whereas others undergo an accelerated ageing process [17]. Hence, individuals of the same chronological age (e.g. the number of years they have been alive) can be biologically (much) younger or older than their birth date may predict, which explains the large heterogeneity in phenotype at older age.

Research suggests that many clinical (age-related) outcomes share this “accelerated biological ageing” as the underlying pathophysiological mechanism for their development, indicating that individuals with an older biological age compared to their chronological age have a high risk of several adverse health outcomes [18, 19]. For this reason, studying age through the lens of chronological age only may be insufficient. Instead, measuring one or more hallmarks of ageing as a measure of biological age may provide valuable novel insights into the relationship between adiposity and the development of CMDs at older age.

In the present study, we aimed to investigate the associations between adiposity and CMDs in different (and sex-specific) groups of chronological age and leukocyte telomere length (LTL) as a measure of biological age using the data from the large prospective cohort study of the UK Biobank [20]. To that end, this study will add additional insights into the previously observed attenuated association between BMI and CMD at an older chronological age.

## Materials and methods

### Study design and population

The UK Biobank cohort is a prospective general population cohort. Baseline measurements were conducted between 2006 and 2010 in 22 different assessment centres across the UK [20]. The UK biobank study was approved by the North-West Multi-centre Research Ethics Committee. Access for information to invite participants was approved by the Patient Information Advisory Group for England and Wales. All participants in the UK Biobank provided a written informed consent. The present project was completed under project number 56340.

In total, 502,628 participants between the ages of 40 and 70 years were enrolled from the general population. This age range was established by the UK Biobank, resulting in a study population old enough for a sufficient number of health outcomes during early follow-up, and young enough before incipient disease had a material impact on exposures [20]. Recruitment was facilitated through invitation letters, sent to all eligible adults registered to the National Health Services (NHS) and living within a distance of 25 miles from one of the study centres. Detailed information on the study design and data collection has been documented previously [20].

For the present study, we restricted the analyses to the UK Biobank participants who reported to be of European ethnicity to prevent influences from the other ancestries in our analyses given the different body compositions of non-European with European-ancestry populations, who had no reported history of CAD, T2D or any stroke at baseline, and who had data on LTL available (*N* = 400,890). The median follow-up time of the included participants was 12 years (for incident CAD, T2D and ischemic stroke).

### Assessment of exposure variables at baseline

Participants of the UK Biobank visited one of the study centres for several baseline measurements, including body composition. Body weight was assessed using the Tanita BC418MA body composition analyser (Tanita, Inc. Manchester, UK). The procedure for body composition measurement by impedance can be found in detail in the UK Biobank protocol for Body Composition Measurement at an Assessment Centre (version 1.0) [21]. In brief, the Tanita BC418MA body composition analyser produces segmental readings of fat percentage, fat (free) mass and predicted muscle mass for the right arm and leg, left arm and leg and trunk. Data are then captured by direct entry into Vox from the Tanita analyser by the staff member. Staff members are healthcare technicians or nurses certified to conduct assessments.

We used BMI, waist-to-hip ratio (WHR)-adjusted-for-BMI, “A body shape index” (ABSI) and total body fat (TBF) as baseline exposures of body shape and adiposity as they are relatively easily measured in standard clinical practice, thus beneficially contributing to the clinical translation of the present study. BMI was calculated using body weight (kg) and height (m). Waist circumference (WC) and hip circumference (HC) were determined by a horizontally positioned tape measure. WC and HC were then used to calculate the WHR, which was subsequently adjusted for BMI prior to any further analyses to get a measure of upper body fat [22]. We calculated the WHR-adjusted-for-BMI by performing a linear regression analysis with WHR as dependent variable and BMI as the independent variable and stored the residuals accordingly [23]. ABSI was calculated using the participant’s WC (m), BMI (kg/m^2^) and weight (kg) at baseline with the following formula [24]: ABSI = $\frac{\mathrm{WC}}{\mathrm{BMI}^\wedge(2/3)\times\mathrm{height}^\wedge(1/2)}$. Total fat mass was measured using the Tanita BC418MA body composition analyser (Tanita, Inc. Manchester, UK), and total fat-free mass was subsequently calculated by subtracting total fat mass from total body weight. TBF was subsequently determined as a percentage of total fat mass (kg) divided by weight (kg).

### Assessment of leukocyte telomere length as potential effect modifier

LTL (µm) was determined in 488,400 participants of the UK Biobank. A detailed description of the LTL measurements is provided elsewhere [25]. Briefly, LTL was measured as the ratio of telomere repeat copy number relative to that of a single copy gene (T/S ratio) using multiplex qPCR methodology. LTL measurements were adjusted for technical variation, log_e_ transformed and *Z*-standardised. We calculated the LTL residual value by performing a linear regression analysis with LTL as the dependent variable and age as the independent variable and stored the residuals accordingly. LTL residuals were arranged in equally sized tertiles for analysis (tertile 1, − 12.69; − 0.41 SD (short); tertile 2, − 0.41–0.41 SD (middle); tertile 3, 0.41–5.92 SD (long)).

### Cardiometabolic disease outcomes

Information on incident cardiometabolic disease was collected through information from the data provided by the NHS record systems. Diagnoses were coded according to the International Classification of Diseases edition 10 (ICD-10) [20]. Here, we defined CAD as a combination of the following diagnoses: angina pectoris (I20; data-field 131,296), myocardial infarction (I21 and I22; data-fields 131,298 and 131,300) and acute and chronic ischemic heart disease (I24 and I25; data-fields 131,304 and 131,306); for the present study, we considered the first date in time in case individuals developed multiple of the diagnoses under the CAD diagnosis. Ischemic stroke was defined as cerebral infarction (I63; data-field 131,366). Prevalent and incident T2D was identified in the UK Biobank as the date of first appearance of non-insulin-dependent diabetes mellitus (E11; data-field 130,708). Composition of the CMD outcome variables CAD, T2D and IS occurred through a standard algorithm which combined the data derived from hospital admissions, general practitioners and death records and through self-report, which was already performed centrally by UK Biobank. Based on the date of first appearance and the data of enrolment, we defined a case as prevalent (before enrolment) or incident (after enrolment). Follow-up time of all CMD outcomes is computed from the baseline visit to the diagnosis of incident disease, loss-to-follow-up or death, or the end of the study period, whichever came first.

### Covariates

The Townsend Deprivation Index (TDI) was based on the preceding national census data on car ownership, household overcrowding, owner occupation and unemployment aggregated for postal codes of residence in the UK. Each participant was assigned a score, with higher TDI scores equating to higher levels of socioeconomic deprivation. The TDI was calculated immediately prior to enrolment of the participant. Systolic and diastolic blood pressure were measured at the study centre using an automated device (Omron device) in resting sitting position. All measurements were performed twice, of which the average systolic and diastolic blood pressure were determined and used for analyses. Information on thyroid status was collected through information from the data provided by the NHS record systems. Thyroid status was determined by the date of first reported iodine-deficiency-related thyroid disorders (ICD-10 code E02), other forms of hypothyroidism (ICD-10 code E03) or thyrotoxicosis (ICD-10 code E05). Based on this data, we classified participants as having hypothyroidism, hyperthyroidism, both or none. Smoking status (“Do you smoke tobacco now?”; yes, only occasionally, no), alcohol use (“How often do you drink alcohol?”; daily or almost daily, three or four times a week, once or twice a week, one to three times a month, special occasions only, never) and blood pressure–lowering and cholesterol-lowering medication use were self-reported via a questionnaire. Participants were additionally asked for their current number of treatments and/or medications during a verbal interview.

Comorbidities were assessed by the date of first reported of a series of ICD-10 codes. We defined two groups of comorbidities: circulatory system disorders (I20, I21, I22, I24, I25, I50, I60, I62, I63, I64, I67, I68, I69, I71, I79) and nervous system disorders (G45, G46). Individuals were coded with a circulatory or nervous system disorder of 1 if the date of first reported ICD-10 code was dated before their entry date into the UK Biobank. Individuals with missing data on circulatory or nervous system disorders, or with a reported date after the entry date into the UK Biobank, were considered free from circulatory or nervous system disorders and coded with 0.

### Statistical analyses

Characteristics of the study population were studied at baseline and expressed as mean (standard deviation, SD), median (interquartile range, IQR; for non-normally distributed data only) or proportion (%) in the whole study population and for men and women separately. All statistical analyses were performed using R (v4.1.2) statistical software (The R Foundation for Statistical Computing, Vienna, Austria).

We performed linear regression analyses to estimate the associations between different measures of body shape and composition at baseline, and chronological age or LTL (adjusted for chronological age) at baseline using linear regression models. Participants were allocated to one of three chronological age groups (≤ 50 years old (young), 51–60 years old (middle-aged), ≥ 61 years old (old)) and one of the three LTL tertiles, of which the young (for chronological age groups) and long (for LTL residuals tertiles) group was used as a reference in the multivariable-adjusted regression analyses. Only the age and LTL at recruitment were considered for these analyses. All regression analyses were conducted for men and women separately, as well as in the total study population.

We additionally performed multivariable-adjusted cox proportional hazard analyses to estimate the associations between different measures of body shape and composition, and CMDs using cox-proportional hazard models implemented in the R-based *survival* package (cran.r-project.org/web/packages/survival). The time scale used for the cox-proportional hazard models was in calendar years. The cox-proportional hazard models were adjusted for sex, systolic blood pressure (mmHg), TDI, thyroid status (normal thyroid function; hypothyroidism; hyperthyroidism), circulatory system disorders, nervous system disorders, smoking status, alcohol use, number of medications taken, blood pressure medication (yes/no) and cholesterol-lowering medication use (yes/no). The cox-proportional hazard models stratified by LTL residuals were additionally adjusted for chronological age (years). To check whether the proportional hazards assumption was fulfilled, a cox-proportional hazard assumption test (“cox.zph” from R package “Survival”) was performed.

Firstly, we associated baseline standardized (mean = 0, standard deviation = 1) measured body composition (notably BMI, ABSI, WHR-adjusted-for-BMI and TBF) with incident CAD, T2D and IS. Secondly, we estimated the association between all baseline exposures and incident CAD, T2D and IS in three different baseline chronological age groups: (i) ≤ 50 years old (young), (ii) 51–60 years old (middle-aged), (iii) ≥ 61 years old (old). Subsequently, we repeated the analyses between baseline exposures and incident CAD, T2D and IS stratified based on the LTL residuals (adjusted for chronological age), in tertiles (tertile 1, − 12.69; − 0.41 SD (short); tertile 2, − 0.41–0.41 SD (middle); tertile 3, 0.41–5.92 SD (long)). All associations were determined for men and women separately, as well as in the total study population.

Additionally, we tested for interaction on a multiplicative scale between the measures of adiposity and chronological age and LTL on CMDs by introducing an interaction term in the fully adjusted cox-proportional hazard model in the total study population, and separately for men and women.

## Results

### Characteristics of the study population

The present study comprised 400,890 participants with a median (IQR) age of 57.0 (50.0–63.0) years, of whom 56% were women (see Table 1). The median (IQR) BMI of the study cohort was 26.5 (24.0–29.6) kg/m^2^. Regarding different age groups, median (IQR) BMI in chronologically young, middle-aged and old individuals was 26.1 (23.5–29.3) kg/m^2^, 26.6 (24.0–29.8) kg/m^2^ and 26.7 (24.3–29.6) kg/m^2^, respectively. Chronologically young individuals had lower median (IQR) waist circumference (87.0 (78.0–96.0 cm)) compared to chronologically old individuals (90.0 (82.0–99.0 cm)), whereas hip circumference was similar in all chronological age groups. Chronologically young individuals also had lower mean (SD) total body fat (29.7 (± 8.57) %) compared to chronologically old individuals (32.2 (± 8.25) %). Median (IQR) LTL in chronologically young individuals was 0.853 (0.77–0.940) µm, compared to 0.798 (0.72–0.880) µm in chronologically old individuals. Results were similar for the different LTL groups, as described in Table 1. In addition, chronological age (years) and LTL (μm) were marginally correlated, with an *R*^2^ of 0.032 (Fig. s1).

**Table 1:** Characteristics of Caucasian UK Biobank study population used for the present study, stratified by chronological age and LTL

	**Young** (40–50 years old) *N* = 107,394	**Middle-aged** (51–60 years old) *N* = 144,720	**Old** (61–70 years old) *N* = 148,776	**Total** *N* = 400,890
**Demography**				
Sex, *n* (%)				
Men	48,042 (45%)	61,713 (43%)	66,564 (45%)	176,319 (44%)
Women	59,352 (55%)	83,007 (57%)	82,212 (55%)	224,571 (56%)
Age (years), median (IQR)	46.0 (43.0–48.0)	56.0 (53.0–58.0)	64.0 (62.0–67.0)	57.0 (50.0–63.0)
Weight (kg), median (IQR)	76.5 (66.0–88.1)	76.1 (66.2–87.3)	75.4 (66.3–85.6)	75.9 (66.2–86.9)
Height (m), mean (SD)	1.70 (± 0.0924)	1.69 (± 0.0918)	1.67 (± 0.0915)	1.69 (± 0.0925)
BMI (kg/m^2^), median (IQR)	26.1 (23.5–29.3)	26.6 (24.0–29.8)	26.7 (24.3–29.6)	26.5 (24.0–29.6)
Waist circumference (cm), median (IQR)	87.0 (78.0–96.0)	89.0 (80.0–98.0)	90.0 (82.0–99.0)	89.0 (80.0–98.0)
Hip circumference, mean (SD)	103 (± 9.16)	103 (± 9.16)	103 (± 8.60)	103 (± 8.96)
Waist-to-hip ratio adjusted for BMI, mean (SD)	− 0.0165 (± 0.0771)	− 0.00546 (± 0.0795)	0.00697 (± 0.0802)	− 0.00379 (± 0.0797)
Body shape index, mean (SD)	0.0751 (± 0.00521)	0.0762 (± 0.00538)	0.0774 (± 0.00545)	0.0763 (± 0.00544)
Fat mass (kg), median (IQR)	21.8 (17.0–28.1)	23.3 (18.3–29.7)	23.7 (18.9–29.6)	23.1 (18.1–29.3)
Total body fat (%), mean (SD)	29.7 (± 8.57)	31.6 (± 8.51)	32.2 (± 8.25)	31.3 (± 8.49)
Systolic blood pressure (mmHg), mean (SD)	130 (± 15.8)	137 (± 17.8)	144 (± 18.7)	138 (± 18.6)
Diastolic blood pressure (mmHg), mean (SD)	81.0 (± 10.3)	83.0 (± 10.1)	82.7 (± 9.81)	82.3 (± 10.1)
Leukocyte telomere length (µm), median (IQR)	0.853 (0.774–0.940)	0.826 (0.750–0.911)	0.798 (0.724–0.880)	0.823 (0.745–0.909)
Townsend Deprivation Index, median (IQR)	− 1.96 (− 3.55–0.749)	− 2.34 (− 3.74–0.0834)	− 2.50 (− 3.81; − 0.269)	− 2.31 (− 3.72–0.139)
Number of medications taken, median (IQR)	1.00 (0–2.00)	1.00 (0–3.00)	2.00 (1.00–4.00)	2.00 (0–3.00)
Thyroid status, *n* (%)				
Hyperthyroidism	3058 (3%)	6860 (5%)	8668 (6%)	18,586 (5%)
Hypothyroidism	3058 (3%)	6860 (5%)	8668 (6%)	18,586 (5%)
Hyper- and hypothyroidism	340 (0%)	652 (0%)	740 (0%)	1732 (0%)
None of the above	103,482 (96%)	136,324 (94%)	138,363 (93%)	378,169 (94%)
Circulatory system disorder, *n* (%)				
Yes	99 (0%)	337 (0%)	683 (0%)	1119 (0%)
No	107,295 (100%)	144,383 (100%)	148,093 (100%)	399,771 (100%)
Nervous system disorder, *n* (%)				
Yes	96 (0%)	435 (0%)	1207 (1%)	1738 (0%)
No	107,298 (100%)	144,285 (100%)	147,569 (99%)	399,152 (100%)
Alcohol use, *n* (%)				
Daily or almost daily	17,638 (16%)	31,344 (22%)	36,416 (24%)	85,398 (21%)
Three or four times a week	26,887 (25%)	37,013 (26%)	33,662 (23%)	97,562 (24%)
Once or twice a week	32,346 (30%)	37,973 (26%)	35,849 (24%)	106,168 (26%)
One to three times a month	14,564 (14%)	15,562 (11%)	14,630 (10%)	44,756 (11%)
Special occasions only	10,446 (10%)	14,273 (10%)	17,025 (11%)	41,744 (10%)
Never	5435 (5%)	8454 (6%)	11,119 (7%)	25,008 (6%)
Prefer not to answer	78 (0%)	101 (0%)	75 (0%)	254 (0%)
Smoking, *n* (%)				
Yes, on most or all days	27,609 (26%)	48,918 (34%)	61,137 (41%)	137,664 (34%)
Only occasionally	14,504 (14%)	15,068 (10%)	11,531 (8%)	41,103 (10%)
No	65,062 (61%)	80,326 (56%)	75,451 (51%)	220,839 (55%)
Prefer not to answer	219 (0%)	408 (0%)	657 (0%)	1284 (0%)
Blood pressure medication, *n* (%)				
Yes	5688 (5%)	21,570 (15%)	39,448 (27%)	66,706 (17%)
No	101,706 (95%)	123,150 (85%)	109,328 (73%)	334,184 (83%)
Cholesterol-lowering medication, *n* (%)				
Yes	3287 (3%)	14,135 (10%)	30,264 (20%)	47,686 (12%)
No	104,107 (97%)	130,585 (90%)	118,512 (80%)	353,204 (88%)
	**Long LTL** (0.876–1.98 μm) *N* = 133,630	**Medium LTL** (0.773–0.876 μm) *N* = 133,630	**Short LTL** (0.129–0.773 μm) *N* = 133,630	**Total** *N* = 400,890
Demography				
Sex, *n* (%)				
Men	51,903 (39%)	59,252 (44%)	65,164 (49%)	176,319 (44%)
Women	81,727 (61%)	74,378 (56%)	68,466 (51%)	224,571 (56%)
Age (years), median (IQR)	58.0 (50.0–63.0)	57.0 (50.0–63.0)	57.0 (50.0–63.0)	57.0 (50.0–63.0)
Weight (kg), median (IQR)	74.6 (65.2–85.6)	76.1 (66.3–87.1)	77.0 (67.0–87.9)	75.9 (66.2–86.9)
Height (m), mean (SD)	1.68 (± 0.0915)	1.69 (± 0.0929)	1.69 (± 0.0928)	1.69 (± 0.0925)
BMI (kg/m^2^), median (IQR)	26.3 (23.8–29.4)	26.6 (24.0–29.6)	26.7 (24.2–29.8)	26.5 (24.0–29.6)
Waist circumference (cm), median (IQR)	88.0 (79.0–97.0)	89.0 (80.0–98.0)	90.0 (81.0–99.0)	89.0 (80.0–98.0)
Hip circumference, mean (SD)	103 (± 8.96)	103 (± 8.98)	103 (± 8.93)	103 (± 8.96)
Waist-to-hip ratio adjusted for BMI, mean (SD)	− 0.00980 (± 0.0788)	− 0.00362 (± 0.0795)	0.00204 (± 0.0803)	− 0.00379 (± 0.0797)
Body shape index, mean (SD)	0.0760 (± 0.00541)	0.0763 (± 0.00543)	0.0766 (± 0.00545)	0.0763 (± 0.00544)
Fat mass (kg), median (IQR)	23.0 (18.1–29.2)	23.1 (18.1–29.4)	23.1 (18.1–29.3)	23.1 (18.1–29.3)
Total body fat (%), mean (SD)	31.7 (± 8.45)	31.3 (± 8.51)	31.0 (± 8.49)	31.3 (± 8.49)
Systolic blood pressure (mmHg), mean (SD)	138 (± 18.7)	138 (± 18.5)	138 (± 18.6)	138 (± 18.6)
Diastolic blood pressure (mmHg), mean (SD)	82.2 (± 10.1)	82.4 (± 10.0)	82.5 (± 10.1)	82.3 (± 10.1)
Leukocyte telomere length (µm), median (IQR)	0.949 (0.907–1.01)	0.823 (0.796–0.850)	0.713 (0.670–0.746)	0.823 (0.745–0.909)
Townsend Deprivation Index, median (IQR)	− 2.34 (− 3.74–0.0568)	− 2.32 (− 3.73–0.121)	− 2.27 (− 3.70–0.245)	− 2.31 (− 3.72–0.139)
Number of medications taken, median (IQR)	1.00 (0–3.00)	2.00 (0–3.00)	2.00 (0–3.00)	2.00 (0–3.00)
Thyroid status, *n* (%)				
Hyperthyroidism	838 (1%)	751 (1%)	814 (1%)	2403 (1%)
Hypothyroidism	6205 (5%)	6209 (5%)	6172 (5%)	18,586 (5%)
Hyper- and hypothyroidism	546 (0%)	593 (0%)	593 (0%)	1732 (0%)
None of the above	126,041 (94%)	126,077 (94%)	126,051 (94%)	378,169 (94%)
Circulatory system disorder, *n* (%)				
Yes	358 (0%)	357 (0%)	404 (0%)	1119 (0%)
No	133,272 (100%)	133,273 (100%)	133,226 (100%)	399,771 (100%)
Nervous system disorder, *n* (%)				
Yes	525 (0%)	586 (0%)	627 (0%)	1738 (0%)
No	133,105 (100%)	133,044 (100%)	133,003 (100%)	399,152 (100%)
Alcohol use, *n* (%)				
Daily or almost daily	27,857 (21%)	28,297 (21%)	29,244 (22%)	85,398 (21%)
Three or four times a week	32,812 (25%)	32,482 (24%)	32,268 (24%)	97,562 (24%)
Once or twice a week	32,503 (24%)	32,526 (24%)	32,533 (24%)	97,562 (24%)
One to three times a month	35,421 (27%)	35,455 (27%)	35,292 (26%)	106,168 (26%)
Special occasions only	15,050 (11%)	14,919 (11%)	14,787 (11%)	44,756 (11%)
Never	8509 (6%)	8359 (6%)	8140 (6%)	25,008 (6%)
Prefer not to answer	79 (0%)	86 (0%)	89 (0%)	254 (0%)
Smoking, *n* (%)				
Yes, on most or all days	45,424 (34%)	45,689 (34%)	46,551 (35%)	137,664 (34%)
Only occasionally	12,177 (9%)	13,476 (10%)	15,450 (12%)	41,103 (10%)
No	75,636 (57%)	74,012 (55%)	71,191 (53%)	220,839 (55%)
Prefer not to answer	393 (0%)	453 (0%)	438 (0%)	1284 (0%)
Blood pressure medication, *n* (%)				
Yes	22,083 (17%)	22,289 (17%)	22,334 (17%)	66,706 (17%)
No	111,547 (83%)	111,341 (83%)	111,296 (83%)	334,184 (83%)
Cholesterol-lowering medication, *n* (%)				
Yes	15,136 (11%)	16,096 (12%)	16,454 (12%)	47,686 (12%)
No	118,494 (89%)	117,534 (88%)	117,176 (88%)	353,204 (88%)

Data are presented as mean (standard deviation) or median (interquartile range) for numerical variables, and number (proportions) for categorical variables

*Missing values:* weight (284 young, 230 middle-aged, 186 old | 221 long LTL, 247 medium LTL, 232 short LTL), height (284 young, 230 middle-aged, 186 old | 221 long LTL, 247 medium LTL, 232 short LTL), Townsend Deprivation Index (169 young, 176 middle-aged, 132 old | 168 long LTL, 166 medium LTL, 143 short LTL), A Body Shape Index (284 young, 230 middle-aged, 186 old | 221 long LTL, 247 medium LTL, 232 short LTL), fat mass (269 young, 218 middle-aged, 185 old | 211 long LTL, 237 medium LTL, 224 short LTL), total body fat (284 young, 230 middle-aged, 186 old | 221 long LTL, 247 medium LTL, 232 short LTL), systolic blood pressure (9497 young, 12,841 middle-aged, 12,112 old | 11,754 long LTL, 11,281 medium LTL, 11,415 short LTL), diastolic blood pressure (9495 young, 12,839 middle-aged, 12,106 old | 11,751 long LTL, 11,279 medium LTL, 11,410 short LTL), number of medications taken (1 young, 2 middle-aged, 4 old | 2 long LTL, 3 medium LTL, 2 short LTL)

### Associations between baseline adiposity measures and chronological age and LTL

Middle chronological age and old chronological age were associated with higher BMI, ABSI, WHR-adjusted-for-BMI and TBF in the total study cohort (BMI, middle: 0.09 SD [95% CI 0.08, 0.10]; BMI, old: 0.09 SD [95% CI 0.08, 0.09]; ABSI, middle: 0.24 SD [95% CI 0.23, 0.24]; ABSI, old: 0.42 SD [95% CI 0.42, 0.43], all compared to chronological age group 1 [≤ 50 years old]), as well as in men and women separately (Tab s1). The association between chronological age and BMI in men, however, attenuated (middle: 0.06 SD [95% CI 0.04, 0.07]; old: 0.01 SD [95% CI − 0.01, 0.02], compared to men of chronological age group 1 [≤ 50 years old]). Similarly, middle and short LTL residuals were associated with higher BMI, ABSI, WHR-adjusted-for-BMI and TBF in the total study cohort, as well as in men and women separately, compared to LTL residuals tertile 3 (Tab s2).

### Associations between baseline adiposity measures and incident CMD in the total study cohort

A one-SD increase in any of the different adiposity measures (e.g. BMI, ABSI, WHR-adjusted-for-BMI and TBF) was associated with a higher risk of incident CAD (26,127 cases), T2D (19,000 cases) and IS (4842 cases) in the total population as well as in men and in women (Table 2).

**Table 2:** Associations between different measures of adiposity and cardiometabolic outcome in the total study population

	CAD	T2D	IS
BMI (kg/m^2^)	1.14 (1.13, 1.16)	1.70 (1.68, 1.72)	1.09 (1.06, 1.12)
ABSI	1.10 (1.08, 1.11)	1.40 (1.37, 1.42)	1.09 (1.06, 1.14)
WHR adj. BMI	1.12 (1.10, 1.14)	1.34 (1.32, 1.37)	1.10 (1.05, 1.15)
TBF (%)	1.19 (1.17, 1.22)	2.29 (2.24, 2.35)	1.10 (1.05, 1.15)

Results presented as the hazard ratio (with 95% confidence interval) per standard deviation increase in the exposure. All hazard ratios are adjusted for age, Townsend Deprivation Index, thyroid status, circulatory system disorders, nervous system disorders, smoking status, alcohol use, systolic blood pressure, number of medications taken, blood pressure medication and cholesterol-lowering medication. CAD: 26,127 events; T2D: 19,000 events; IS: 4842 events

*ABSI*, a body shape index; *BMI*, body mass index; *CAD*, coronary artery disease; *IS*, ischemic stroke; *TBF*, total body fat; *T2D*, type 2 diabetes; *WHR*, waist-to-hip ratio

### Associations between baseline adiposity measures and incident CAD, T2D and IS with increasing chronological age

**Fig. 1:**
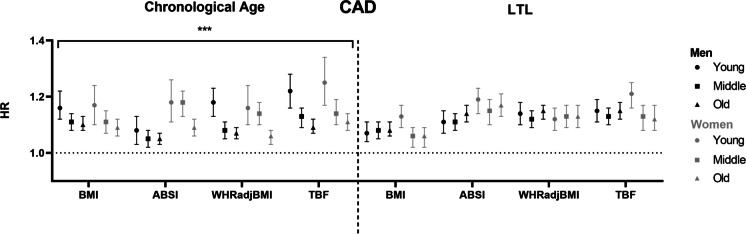
Associations between different measures of adiposity and cardiometabolic outcome in men and women, stratified by chronological age or LTL. (Left panel) Hazard ratios are shown per age group (young: ≤ 50 years old, middle: 51–60 years old, old: ≥ 61 years old) in men (black) and women (grey). (Right panel) Hazard ratios are shown per tertile LTL residuals (long: 0.41–5.92 SD, middle: − 0.41–0.41 SD, short: − 12.69; − 0.41 SD) in men (black) and women (grey). *Abbreviations:* ABSI, a body shape index; BMI, body mass index; CAD, coronary artery disease; HR, hazard ratio; LTL, leukocyte telomere length; TBF, total body fat; WHR, waist-to-hip ratio

**Fig. 2:**
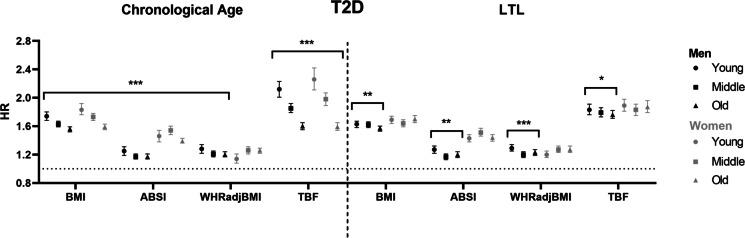
Associations between different measures of adiposity and cardiometabolic outcome in men and women, stratified by chronological age or LTL. (Left panel) Hazard ratios are shown per age group (young: ≤ 50 years old, middle: 51–60 years old, old: ≥ 61 years old) in men (black) and women (grey). (Right panel) Hazard ratios are shown per tertile LTL residuals (long: 0.41–5.92 SD, middle: − 0.41–0.41 SD, short: − 12.69; − 0.41 SD) in men (black) and women (grey). *Abbreviations:* ABSI, a body shape index; BMI, body mass index; HR, hazard ratio; LTL, leukocyte telomere length; TBF, total body fat; T2D, type 2 diabetes; WHR, waist-to-hip ratio

**Fig. 3:**
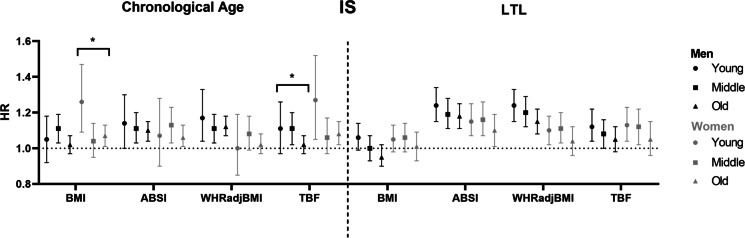
Associations between different measures of adiposity and cardiometabolic outcome in men and women, stratified by chronological age or LTL. (Left panel) Hazard ratios are shown per age group (young: ≤ 50 years old, middle: 51–60 years old, old: ≥ 61 years old) in men (black) and women (grey). (Right panel) Hazard ratios are shown per tertile LTL residuals (long: 0.41–5.92 SD, middle: − 0.41–0.41 SD, short: − 12.69; − 0.41 SD) in men (black) and women (grey). *Abbreviations:* ABSI, a body shape index; BMI, body mass index; HR, hazard ratio; IS, ischemic stroke; LTL, leukocyte telomere length; TBF, total body fat; WHR, waist-to-hip ratio

We observed an association between higher baseline adiposity measures BMI, ABSI, WHR-adjusted-for-BMI and TBF, and higher HR for incident CAD and T2D in all three chronological age groups, in men as well as in women (Figs. 1 and 2, left panel) (Tab s3 and s4). The risk of incident IS was higher in some, but not all observed associations with baseline BMI, ABSI, WHR-adjusted-for-BMI and TBF in men and women (Fig. 3, left panel) (Tab s3 and s4). Similar associations were found in the total study population (Tab s5).

Notably, the associations between all baseline exposures and incident CAD and T2D attenuated with increasing chronological age (all *P*_interactions_ < 0.001) (Figs. 1 and 2, left panel) (Tab s3-s5), with the exception of the association between WHR-adjusted-for-BMI and T2D in women. The youngest group, aged 50 years and younger at baseline, had a higher risk of developing incident CAD with a higher BMI than the oldest group, who were aged 61 years an older at baseline (young men: HR 1.16 [95% CI 1.12, 1.22] per SD; old men: HR 1.10 [95% CI 1.08, 1.13] per SD; young women: HR 1.17 [95% CI 1.10, 1.24] per SD; old women: HR 1.09 [95% CI 1.06, 1.12] per SD) (all *P*_interaction_ < 0.001). A similar attenuation of the risk of incident CAD was observed for higher ABSI, WHR-adjusted-for-BMI and TBF in both men and women. In addition, the youngest group had a higher risk of developing incident T2D with higher BMI than the oldest group (young men: HR 1.74 [95% CI 1.68, 1.80] per SD BMI; old men: HR 1.56 [95% CI 1.52, 1.59] per SD BMI; young women: HR 1.83 [95% CI 1.76, 1.92] per SD BMI; old women: HR 1.59 [95% CI 1.55, 1.63] per SD BMI) (all *P*_interaction_ < 0.001). In men, similar results were observed for the association between ABSI, WHR-adjusted-for-BMI and TBF, and incident T2D. In women, similar results were observed for the associations between ABSI and TBF, but not for WHR-adjusted-for-BMI.

### Associations between baseline adiposity measures and incident CAD, T2D and IS with decreasing LTL residuals

Upon stratification by LTL residuals, we did not observe effect modification of LTL on the association between BMI and incident CAD in men as well as in women (long LTL in men: HR 1.07 [95% CI 1.04, 1.11] per SD; short LTL in men: HR 1.08 [95% CI 1.06, 1.11] per SD; long LTL in women: HR 1.13 [95% CI 1.09, 1.17] per SD; short LTL in women: HR 1.06 [95% CI 1.02, 1.09] per SD) (*P*_interaction_ men = 0.85; *P*_interaction_ women = 0.27) (Fig. 1, right panel) (Tab s6 and s7). Similar results were observed for baseline adiposity measures ABSI, WHR-adjusted-for-BMI and TBF, and incident CAD. In contrast, we observed effect modification of LTL on the association between BMI, ABSI, WHR-adjusted-for-BMI and TBF, and incident T2D in men (BMI, long LTL: HR 1.63 [95% CI 1.58, 1.67] per SD; BMI, short LTL: HR 1.57 [95% CI 1.53, 1.60]) per SD) (*P*_interaction_ = 0.005) (Fig. 2, right panel) (Tab s6), although the observed effects were smaller than the effect modification observed for chronological age. These results for incident T2D were not observed in women. Regarding IS, we did not observe any effect of LTL on the association between BMI, ABSI, WHR-adjusted-for-BMI and TBF, and incident T2D and IS in both men and women (Fig. 3, right panel) (Tab s6 and s7). Similar results were found in the total study population (Tab s8), and for the associations between measures of adiposity, and incident CAD, T2D and IS upon stratification by LTL in micrometres (Tab s9-s11).

## Discussion

For the present study, we aimed to investigate the (biological) age-dependent association between different baseline measures of body shape and composition, and incident cardiometabolic diseases in participants of the UK Biobank. We observed that higher baseline BMI, ABSI, WHR-adjusted-for-BMI and TBF were associated with higher risks for incident CAD and T2D in men and women upon stratification by chronological and (chronological age-adjusted) LTL as a measure for biological age. Importantly, whereas the associations between adiposity measures and incident CMD risk attenuated with increasing chronological age, we did not observe an attenuation with decreasing (chronological age-adjusted) LTL in men nor women.

The attenuation of the association between adiposity measures and CMDs with increasing chronological age has been observed in previous studies. For example, a recent MR study using data from the UK Biobank showed that the associations between the genetically influenced exposures for cardiovascular risk factors (e.g. BMI, low-density lipoprotein cholesterol, triglycerides and high blood pressure) and primary CAD attenuated with increasing age, indicating that the causal risk of primary CAD by classical risk factors is (chronological) age dependent [8]. In addition, an MR study observed that obesity-related traits were less strongly associated with T2D diagnosis at older age compared to younger age in participants of the UK Biobank [9].

However, most previous studies only consider BMI as a measure of (overall) adiposity. Considering that BMI only takes into account weight and height, we investigated the associations between several measures of body shape and adiposity, and incident cardiometabolic disease to provide a more comprehensive perspective of CMD research. In addition, the aforementioned MR studies only considered the participants’ chronological age and did thus not study the effect of heterogeneity in biological age on the associations between adiposity and CMD development. Since the biological age holds considerably more information about an individual’s health and disease status than chronological age, it is an important factor to take into account when studying the age-dependent CMD risk. One of the hallmarks of ageing, telomere attrition [15], may provide a method to address biological age. Telomere length (TL) is a key determinant of proliferative capacity and cellular lifespan which triggers cellular senescence once a critically short telomere length is reached [26]. Regarding that TL, commonly measured in leukocytes (LTL), shows a consistent negative association with age in several studies [27–29], LTL has been proposed as a marker for biological age [30].

The discrepancy between the observed associations between adiposity measures and incident CMD upon stratification by chronological or LTL residuals may be explained by heterogeneity in underlying ageing mechanisms that are not accounted for with chronological age. Age is an important determinant of cardiometabolic health as it is associated with multiple pathological alterations in cardiovascular tissue structure and functions, leading to for example insufficient vascular growth and loss of adequate tissue perfusion [31]. In fact, accelerated tissue ageing has been associated with increased adiposity due to lower basal metabolic rate and reduced physical activity at older age [32]. This adult weight gain is associated with relatively more visceral adipose tissue (VAT) than abdominal subcutaneous adipose tissue (aSAT) [33]. The redistribution of adipose tissue may be the result of the limited capacity of aSAT to expand and store lipids. Considering that the number of adipocytes was shown to remain constant after the age of 20 [34], adipocytes can only increase in size in older individuals, leading to a ‘spill over’ of excess lipids from the subcutaneous to the visceral compartment. Excess VAT is, however, detrimental for health as it is strongly associated with insulin resistance and T2D [33].

This increased visceral adiposity at older age may induce a chronic state of low-grade inflammation, a process known as *inflammaging* [35, 36], as a result of increased secretion of cytokines and fatty acids by adipocytes. For example, VAT is believed to be an important source of inflammatory cytokine interleukin-6 (IL-6) [37], of which the production is enhanced in obese individuals [38]. Furthermore, age-related changes in adipose tissue were associated with increased proinflammatory marker tumour necrosis factor-alpha (TNF-α) [32, 39, 40]. The synthesis of these proinflammatory cytokines is generally inhibited by the anti-inflammatory adipokine adiponectin [32]. However, a recent study observed that the association between VAT and insulin resistance is only mediated by adiponectin to a small extent, suggesting that there are additional contributing factors in the association between VAT and insulin resistance [41]. Taken together, the discrepancy in the associations between adiposity measures and incident CMDs with increasing chronological or decreasing LTL as a measure of biological age that we observed in the present study may (in part) be explained by age-associated changes in adiposity and the proinflammatory features of increased adipose tissue.

Although the present study was conducted in a large study population with a relatively large number of primary CAD and IS, and T2D cases, some limitations should be addressed. Firstly, although TL is a well-studied determinant of proliferative capacity and cellular lifespan [26], using a single measure of LTL per se as a measure of biological age may only allow a rough estimate of an individual’s ageing rate since LTL may depend on transient adaptations in the immune system that are not necessarily related to ageing (e.g. in inflammatory conditions) [30, 42]. The enhanced leukocyte turnover after induction of an immune response may influence the average LTL since newly released leukocytes have longer telomere lengths than mature leukocytes [42]. However, despite its limitations, LTL is still commonly used as a conventional biomarker of ageing as telomeres are critically implicated in cellular ageing [30], and because LTL is relatively easy to measure with limited invasiveness [25]. Moreover, a recent study investigating TL across tissues showed that TL measured in leukocytes from whole blood was a proxy for TL in many tissues, including lung, pancreas, brain and nerve tissue, and that relative TL was negatively associated with chronological age in 21 tissue types [43].

Secondly, we limited our analyses to healthy individuals of European ethnicity with regard to the heterogeneity in the non-European-ancestry sample of the UK Biobank. Translation of our findings to other ancestry groups should therefore be conducted with caution. Third, the Tanita bioimpedance analysis for body composition may not reflect the golden standard for body composition measurement, the dual energy X-ray absorptiometry (DEXA) body scan [44]. However, research has shown bioimpedance analysis to be a valid tool for the assessments of total body and segmental body composition when compared to the DEXA method [45]. Lastly, the data stratified by chronological age may be affected by recall and selection bias due to the participants’ self-reported medication use and the sampling population of “healthy volunteers” in the UK Biobank, respectively.

In summary, the results of the present study showed that higher baseline BMI, ABSI, WHR-adjusted-for-BMI and TBF are associated with higher incident CAD and T2D, but not with higher incident IS. This association was largest at young chronological age and attenuated with increasing chronological age; no attenuation in effect sizes was observed with decreasing LTL as a measure of increasing biological age. When validated in independent samples and with other measures reflecting the biological age, these results could provide valuable information for age-specific recommendations for CMD disease prevention.

## Supplementary Information

Below is the link to the electronic supplementary material.

Supplementary file 1 (DOCX 63 kb)

Supplementary file 2 (PNG 328 kb)

High resolution image (TIFF 401 kb)

## Data Availability

Data of the UK Biobank is available upon acceptance of a research proposal submitted to UK Biobank Resources (https://www.ukbiobank.ac.uk/).
