# Supplementary material for: The association of measures of body shape and adiposity with incidence of cardiometabolic disease from an ageing perspective
Source: GeroScience. 2022 Sep 21;45(1):463–76. doi: 10.1007/s11357-022-00654-9 (PMC9886769; doi:10.1007/s11357-022-00654-9)
Supplement: Supplementary file 1 — (DOCX 63 kb) [file 11357_2022_654_MOESM1_ESM.docx]

**Supplementary Tables**

**Table s1. Associations between different measures of adiposity and chronological age**

|  | **Total** | | **Men** | | **Women** | |
| --- | --- | --- | --- | --- | --- | --- |
|  | *Middle* | *Old* | *Middle* | *Old* | *Middle* | *Old* |
| **BMI (kg/m^2^)** | 0.088  (0.080, 0.096) | 0.086  (0.079, 0.094) | 0.055  (0.044, 0.067) | 0.005  (-0.007, 0.017) | 0.111  (0.100, 0.121) | 0.142  (0.131, 0.152) |
| **ABSI** | 0.236  (0.230, 0.243) | 0.424  (0.418, 0.431) | 0.343  (0.332, 0.355) | 0.606  (0.595, 0.617) | 0.241  (0.231, 0.252) | 0.440  (0.430, 0.451) |
| **WHR adj. BMI** | 0.167  (0.162, 0.173) | 0.294  (0.288, 0.300) | 0.308  (0.296, 0.319) | 0.503  (0.492, 0.515) | 0.184  (0.174, 0.195) | 0.348  (0.337, 0.358) |
| **TBF (%)** | 0.194  (0.188, 0.200) | 0.292  (0.286, 0.298) | 0.253  (0.242, 0.265) | 0.411  (0.400, 0.423) | 0.265  (0.255, 0.276) | 0.381  (0.371, 0.391) |

Results are derived from linear regression coefficients (with 95% confidence interval) per standard deviation increase in the exposure. Chronologically young participants (≤ 50 y/o) were used as a reference group.

*Abbreviations:* ABSI, a body shape index; BMI, body mass index; TBF, total body fat; WHR, waist-to-hip ratio.

**Table s2. Associations between different measures of adiposity and leukocyte telomere length**

|  | **Total** | | **Men** | | **Women** | |
| --- | --- | --- | --- | --- | --- | --- |
|  | *Middle* | *Short* | *Middle* | *Short* | *Middle* | *Short* |
| **BMI (kg/m^2^)** | 0.045  (0.037, 0.053) | 0.069  (0.062, 0.077) | 0.048  (0.036, 0.059) | 0.060  (0.048, 0.072) | 0.043  (0.033, 0.053) | 0.079  (0.069, 0.089) |
| **ABSI** | 0.001  (-0.005, 0.007) | 0.011  (0.005, 0.017) | 0.003  (-0.009, 0.014) | 0.017  (0.005, 0.028) | 0.001  (-0.009, 0.010) | 0.010  (0.001, 0.020) |
| **WHR adj. BMI** | 0.001  (-0.005, 0.006) | 0.008  (0.003, 0.014) | 0.007  (-0.005, 0.018) | 0.030  (0.018, 0.041) | -0.002  (-0.012, 0.008) | -0.001  (-0.012, 0.009) |
| **TBF (%)** | 0.032  (0.026, 0.037) | 0.056  (0.050, 0.061) | 0.043  (0.031, 0.055) | 0.072  (0.060, 0.084) | 0.042  (0.032, 0.052) | 0.077  (0.067, 0.087) |

Results are derived from linear regression coefficients (with 95% confidence interval) per standard deviation increase in the exposure. Participants with the highest LTL residuals (tertile 1; 0.41 – 5.91 SD) were used as a reference group.

*Abbreviations:* ABSI, a body shape index; BMI, body mass index; TBF, total body fat; WHR, waist-to-hip ratio

**Table s3. Associations between different measures of adiposity and cardiometabolic outcome in men, stratified by chronological age**

|  |  | **Men** | | |
| --- | --- | --- | --- | --- |
|  | *n* | CAD | T2D | IS |
| **BMI (kg/m^2^)** |  |  |  |  |
| ≤ 50 y/o | 48,042 | 1.16 (1.12, 1.22) | 1.74 (1.68, 1.80) | 1.05 (0.92, 1.18) |
| 51-60 y/o | 61,713 | 1.11 (1.08, 1.14) | 1.63 (1.59, 1.67) | 1.11 (1.03, 1.19) |
| ≥ 61 y/o | 66,564 | 1.10 (1.08, 1.13) | 1.56 (1.52, 1.59) | 1.02 (0.97, 1.07) |
| **ABSI** |  |  |  |  |
| ≤ 50 y/o | 47,776 | 1.08 (1.03, 1.13) | 1.25 (1.19, 1.31) | 1.14 (1.00, 1.30) |
| 51-60 y/o | 61,502 | 1.05 (1.02, 1.08) | 1.17 (1.14, 1.21) | 1.11 (1.03, 1.20) |
| ≥ 61 y/o | 66,398 | 1.05 (1.03, 1.07) | 1.17 (1.13, 1.20) | 1.10 (1.04, 1.15) |
| **WHR adj. BMI** |  |  |  |  |
| ≤ 50 y/o | 48,042 | 1.18 (1.13, 1.23) | 1.28 (1.22, 1.34) | 1.17 (1.04, 1.33) |
| 51-60 y/o | 61,713 | 1.08 (1.05, 1.11) | 1.21 (1.17, 1.25) | 1.11 (1.03, 1.19) |
| ≥ 61 y/o | 66,564 | 1.07 (1.05, 1.09) | 1.20 (1.17, 1.24) | 1.12 (1.07, 1.18) |
| **TBF (%)** |  |  |  |  |
| ≤ 50 y/o | 47,776 | 1.22 (1.16, 1.28) | 2.12 (2.01, 2.23) | 1.11 (0.97, 1.26) |
| 51-60 y/o | 61,502 | 1.13 (1.09, 1.16) | 1.85 (1.79, 1.92) | 1.11 (1.02, 1.20) |
| ≥ 61 y/o | 66,398 | 1.09 (1.07, 1.12) | 1.60 (1.55, 1.65) | 1.02 (0.97, 1.07) |

Resulted presented as the hazard ratio (with 95% confidence interval). All hazard ratios are adjusted for age, Townsend Deprivation Index, thyroid status, circulatory system disorders, nervous system disorders, smoking status, alcohol use, systolic blood pressure, number of medications taken, blood pressure medication and cholesterol-lowering medication. CAD: 26,127 events; T2D: 19,000 events; IS: 4,842 events.

*Abbreviations:* ABSI, a body shape index; BMI, body mass index; CAD, coronary artery disease; IS, ischemic stroke; TBF, total body fat; T2D, type 2 diabetes; WHR, waist-to-hip ratio; y/o, year old.

**Table s4. Associations between different measures of adiposity and cardiometabolic outcome in women, stratified by chronological age**

|  |  | **Women** | | |
| --- | --- | --- | --- | --- |
|  | *n* | CAD | T2D | IS |
| **BMI (kg/m^2^)** |  |  |  |  |
| ≤ 50 y/o | 59,352 | 1.17 (1.10, 1.24) | 1.83 (1.76, 1.92) | 1.26 (1.09, 1.47) |
| 51-60 y/o | 83,007 | 1.11 (1.07, 1.15) | 1.73 (1.68, 1.78) | 1.04 (0.95, 1.14) |
| ≥ 61 y/o | 82,212 | 1.09 (1.06, 1.12) | 1.59 (1.55, 1.63) | 1.07 (1.01, 1.13) |
| **ABSI** |  |  |  |  |
| ≤ 50 y/o | 59,334 | 1.18 (1.11, 1.26) | 1.46 (1.38, 1.54) | 1.07 (0.90, 1.28) |
| 51-60 y/o | 82,988 | 1.18 (1.13, 1.22) | 1.54 (1.48, 1.60) | 1.13 (1.03, 1.23) |
| ≥ 61 y/o | 82,192 | 1.09 (1.06, 1.12) | 1.39 (1.36, 1.43) | 1.06 (1.01, 1.13) |
| **WHR adj. BMI** |  |  |  |  |
| ≤ 50 y/o | 59,352 | 1.16 (1.10, 1.24) | 1.14 (1.08, 1.21) | 1.00 (0.85, 1.19) |
| 51-60 y/o | 83,007 | 1.14 (1.10, 1.18) | 1.26 (1.21, 1.31) | 1.08 (0.99, 1.18) |
| ≥ 61 y/o | 82,212 | 1.06 (1.03, 1.08) | 1.26 (1.22, 1.29) | 1.02 (0.97, 1.08) |
| **TBF (%)** |  |  |  |  |
| ≤ 50 y/o | 59,334 | 1.25 (1.17, 1.34) | 2.26 (2.11, 2.42) | 1.27 (1.05, 1.52) |
| 51-60 y/o | 82,988 | 1.14 (1.10, 1.19) | 1.98 (1.89, 2.07) | 1.06 (0.97, 1.17) |
| ≥ 61 y/o | 82,192 | 1.11 (1.08, 1.14) | 1.59 (1.54, 1.65) | 1.08 (1.02, 1.15) |

Resulted presented as the hazard ratio (with 95% confidence interval). All hazard ratios are adjusted for age, Townsend Deprivation Index, thyroid status, circulatory system disorders, nervous system disorders, smoking status, alcohol use, systolic blood pressure, number of medications taken, blood pressure medication and cholesterol-lowering medication. CAD: 26,127 events; T2D: 19,000 events; IS: 4,842 events.

*Abbreviations:* ABSI, a body shape index; BMI, body mass index; CAD, coronary artery disease; IS, ischemic stroke; TBF, total body fat; T2D, type 2 diabetes; WHR, waist-to-hip ratio; y/o, year old.

**Table s5. Associations between different measures of adiposity and cardiometabolic outcome in the total study population, stratified by chronological age**

|  |  | **Total** | | |
| --- | --- | --- | --- | --- |
|  | *n* | CAD | T2D | IS |
| **BMI (kg/m^2^)** |  |  |  |  |
| ≤ 50 y/o | 107,394 | 1.19 (1.15, 1.23) | 1.82 (1.77, 1.87) | 1.15 (1.04, 1.26) |
| 51-60 y/o | 144,720 | 1.12 (1.09, 1,14) | 1.70 (1.67, 1.74) | 1.09 (1.03, 1.15) |
| ≥ 61 y/o | 148,776 | 1.10 (1.08, 1.12) | 1.59 (1.56, 1.61) | 1.04 (1.00, 1.08) |
| **ABSI** |  |  |  |  |
| ≤ 50 y/o | 107,110 | 1.16 (1.10, 1.21) | 1.44 (1.38, 1.51) | 1.14 (1.01, 1.30) |
| 51-60 y/o | 144,490 | 1.13 (1.10, 1.16) | 1.43 (1.39, 1.48) | 1.15 (1.07, 1.23) |
| ≥ 61 y/o | 148,590 | 1.09 (1.06, 1.11) | 1.36 (1.32, 1.39) | 1.10 (1.05, 1.15) |
| **WHR adj. BMI** |  |  |  |  |
| ≤ 50 y/o | 107,394 | 1.26 (1.20, 1.33) | 1.30 (1.23, 1.37) | 1.15 (1.00, 1.33) |
| 51-60 y/o | 144,720 | 1.16 (1.12, 1.20) | 1.35 (1.30, 1.40) | 1.14 (1.05, 1.23) |
| ≥ 61 y/o | 148,776 | 1.09 (1.07, 1.12) | 1.34 (1.30, 1.38) | 1.10 (1.05, 1.16) |
| **TBF (%)** |  |  |  |  |
| ≤ 50 y/o | 107,110 | 1.34 (1.28, 1.42) | 2.89 (2.73, 3.06) | 1.24 (1.07, 1.44) |
| 51-60 y/o | 144,490 | 1.20 (1.16, 1.24) | 2.46 (2.37, 2.56) | 1.13 (1.04, 1.23) |
| ≥ 61 y/o | 148,590 | 1.14 (1.11, 1.17) | 1.93 (1.87, 2.00) | 1.07 (1.01, 1.12) |

Resulted presented as the hazard ratio (with 95% confidence interval). All hazard ratios are adjusted for age, Townsend Deprivation Index, thyroid status, circulatory system disorders, nervous system disorders, smoking status, alcohol use, systolic blood pressure, number of medications taken, blood pressure medication and cholesterol-lowering medication. CAD: 26,127 events; T2D: 19,000 events; IS: 4,842 events.

*Abbreviations:* ABSI, a body shape index; BMI, body mass index; CAD, coronary artery disease; IS, ischemic stroke; TBF, total body fat; T2D, type 2 diabetes; WHR, waist-to-hip ratio; y/o, year old.

**Table s6. Associations between different measures of adiposity and cardiometabolic outcome in men, stratified by leukocyte telomere length residuals**

|  |  | **Men** | | |
| --- | --- | --- | --- | --- |
|  | *n* | CAD | T2D | IS |
| **BMI (kg/m^2^)** |  |  |  |  |
| Long | 51,903 | 1.07 (1.04, 1.11) | 1.63 (1.58, 1.67) | 1.06 (0.99, 1.14) |
| Middle | 59,252 | 1.08 (1.05, 1.11) | 1.62 (1.58, 1.66) | 1.00 (0.93, 1.07) |
| Short | 65,164 | 1.08 (1.06, 1.11) | 1.57 (1.53, 1.60) | 0.95 (0.90, 1.02) |
| **ABSI** |  |  |  |  |
| Long | 51,710 | 1.11 (1.07, 1.15) | 1.27 (1.22, 1.32) | 1.24 (1.15, 1.34) |
| Middle | 59,024 | 1.11 (1.08, 1.14) | 1.17 (1.13, 1.21) | 1.19 (1.11, 1.28) |
| Short | 64,942 | 1.14 (1.11, 1.17) | 1.20 (1.16, 1.24) | 1.18 (1.11, 1.25) |
| **WHR adj. BMI** |  |  |  |  |
| Long | 51,903 | 1.14 (1.10, 1.18) | 1.29 (1.25, 1.34) | 1.24 (1.15, 1.33) |
| Middle | 59,252 | 1.12 (1.09, 1.15) | 1.20 (1.16, 1.24) | 1.20 (1.12, 1.29) |
| Short | 65,164 | 1.15 (1.12, 1.17) | 1.23 (1.19, 1.27) | 1.15 (1.08, 1.22) |
| **TBF (%)** |  |  |  |  |
| Long | 51,710 | 1.15 (1.11, 1.19) | 1.83 (1.76, 1.91) | 1.12 (1.04, 1.22) |
| Middle | 59,024 | 1.13 (1.10, 1.16) | 1.79 (1.73, 1.86) | 1.08 (1.00, 1.16) |
| Short | 64,942 | 1.15 (1.12, 1.18) | 1.76 (1.71, 1.82) | 1.05 (0.98, 1.12) |

Resulted presented as the hazard ratio (with 95% confidence interval). All hazard ratios are adjusted for age, Townsend Deprivation Index, thyroid status, circulatory system disorders, nervous system disorders, smoking status, alcohol use, systolic blood pressure, number of medications taken, blood pressure medication and cholesterol-lowering medication. CAD: 26,127 events; T2D: 19,000 events; IS: 4,842 events.

*Abbreviations:* ABSI, a body shape index; BMI, body mass index; CAD, coronary artery disease; IS, ischemic stroke; TBF, total body fat; T2D, type 2 diabetes; WHR, waist-to-hip ratio.

**Table s7. Associations between different measures of adiposity and cardiometabolic outcome in women, stratified by leukocyte telomere length residuals**

|  |  | **Women** | | |
| --- | --- | --- | --- | --- |
|  | *n* | CAD | T2D | IS |
| **BMI (kg/m^2^)** |  |  |  |  |
| Long | 81,727 | 1.13 (1.09, 1.17) | 1.69 (1.64, 1.74) | 1.05 (0.98, 1.13) |
| Middle | 74,378 | 1.06 (1.02, 1.09) | 1.64 (1.59, 1.69) | 1.06 (0.98, 1.14) |
| Short | 68,466 | 1.06 (1.02, 1.09) | 1.70 (1.65, 1.75) | 1.01 (0.93, 1.09) |
| **ABSI** |  |  |  |  |
| Long | 81,699 | 1.19 (1.14, 1.23) | 1.43 (1.38, 1.48) | 1.15 (1.07, 1.25) |
| Middle | 74,359 | 1.15 (1.10, 1.19) | 1.51 (1.46, 1.57) | 1.16 (1.07, 1.26) |
| Old | 68,456 | 1.17 (1.13, 1.21) | 1.44 (1.39, 1.48) | 1.10 (1.01, 1.19) |
| **WHR adj. BMI** |  |  |  |  |
| Long | 81,727 | 1.12 (1.08, 1.16) | 1.20 (1.16, 1.25) | 1.10 (1.02, 1.18) |
| Middle | 74,378 | 1.13 (1.09, 1.17) | 1.27 (1.23, 1.32) | 1.11 (1.03, 1.20) |
| Short | 68,466 | 1.13 (1.09, 1.17) | 1.27 (1.23, 1.32) | 1.04 (0.96, 1.12) |
| **TBF (%)** |  |  |  |  |
| Long | 81,699 | 1.21 (1.16, 1.25) | 1.89 (1.81, 1.98) | 1.13 (1.04, 1.23) |
| Middle | 74,359 | 1.13 (1.08, 1.17) | 1.83 (1.75, 1.91) | 1.12 (1.02, 1.22) |
| Short | 68,456 | 1.12 (1.08, 1.17) | 1.87 (1.79, 1.96) | 1.05 (0.96, 1.15) |

Resulted presented as the hazard ratio (with 95% confidence interval). All hazard ratios are adjusted for age, Townsend Deprivation Index, thyroid status, circulatory system disorders, nervous system disorders, smoking status, alcohol use, systolic blood pressure, number of medications taken, blood pressure medication and cholesterol-lowering medication. CAD: 26,127 events; T2D: 19,000 events; IS: 4,842 events.

*Abbreviations:* ABSI, a body shape index; BMI, body mass index; CAD, coronary artery disease; IS, ischemic stroke; TBF, total body fat; T2D, type 2 diabetes; WHR, waist-to-hip ratio.

**Table s8. Associations between different measures of adiposity and cardiometabolic outcome in the total study population, stratified by leukocyte telomere length residuals**

|  |  | **Total** | | |
| --- | --- | --- | --- | --- |
|  | *n* | CAD | T2D | IS |
| **BMI (kg/m^2^)** |  |  |  |  |
| Long | 133,630 | 1.11 (1.08, 1.13) | 1.68 (1.65, 1.71) | 1.06 (1.01, 1.12) |
| Middle | 133,630 | 1.08 (1.06, 1.10) | 1.65 (1.62, 1.68) | 1.03 (0.98, 1.09) |
| Short | 133,630 | 1.09 (1.06, 1.11) | 1.64 (1.61, 1.68) | 0.98 (0.93, 1.03) |
| **ABSI** |  |  |  |  |
| Long | 133,409 | 1.18 (1.15, 1.22) | 1.44 (1.40, 1.49) | 1.24 (1.16, 1.32) |
| Middle | 133,383 | 1.16 (1.13, 1.19) | 1.41 (1.36, 1.45) | 1.22 (1.15, 1.30) |
| Short | 133,398 | 1.19 (1.16, 1.22) | 1.40 (1.36, 1.43) | 1.18 (1.11, 1.25) |
| **WHR adj. BMI** |  |  |  |  |
| Long | 133,630 | 1.19 (1.15, 1.23) | 1.35 (1.31, 1.40) | 1.22 (1.14, 1.32) |
| Middle | 133,630 | 1.18 (1.15, 1.22) | 1.35 (1.30, 1.39) | 1.23 (1.14, 1.32) |
| Short | 133,630 | 1.21 (1.17, 1.24) | 1.37 (1.33, 1.42) | 1.15 (1.07, 1.23) |
| **TBF (%)** |  |  |  |  |
| Long | 133,409 | 1.15 (1.21, 1.29) | 2.33 (2.24, 2.43) | 1.18 (1.09, 1.28) |
| Middle | 133,383 | 1.19 (1.15, 1.23) | 2.27 (2.18, 2.36) | 1.14 (1.05, 1.23) |
| Short | 133,398 | 1.20 (1.17, 1.24) | 2.28 (2.19, 2.37) | 1.07 (1.00, 1.15) |

Resulted presented as the hazard ratio (with 95% confidence interval). All hazard ratios are adjusted for age, Townsend Deprivation Index, thyroid status, circulatory system disorders, nervous system disorders, smoking status, alcohol use, systolic blood pressure, number of medications taken, blood pressure medication and cholesterol-lowering medication. CAD: 26,127 events; T2D: 19,000 events; IS: 4,842 events.

*Abbreviations:* ABSI, a body shape index; BMI, body mass index; CAD, coronary artery disease; IS, ischemic stroke; TBF, total body fat; T2D, type 2 diabetes; WHR, waist-to-hip ratio.

**Table s9. Associations between different measures of adiposity and cardiometabolic outcome in men, stratified by leukocyte telomere length**

|  |  | **Men** | | |
| --- | --- | --- | --- | --- |
|  | *n* | CAD | T2D | IS |
| **BMI (kg/m^2^)** |  |  |  |  |
| Long | 51,903 | 1.08 (1.04, 1.11) | 1.65 (1.60, 1.70) | 1.05 (0.98, 1.13) |
| Middle | 59,252 | 1.07 (1.04, 1.09) | 1.62 (1.58, 1.66) | 1.01 (0.94, 1.09) |
| Short | 65,164 | 1.10 (1.07, 1.13) | 1.55 (1.52, 1.59) | 0.97 (0.90, 1.03) |
| **ABSI** |  |  |  |  |
| Long | 51,710 | 1.12 (1.08, 1.16) | 1.28 (1.23, 1.33) | 1.24 (1.14, 1.34) |
| Middle | 59,024 | 1.12 (1.09, 1.15) | 1.16 (1.12, 1.20) | 1.23 (1.15, 1.32) |
| Short | 64,942 | 1.11 (1.08, 1.14) | 1.19 (1.15, 1.24) | 1.15 (1.08, 1.22) |
| **WHR adj. BMI** |  |  |  |  |
| Long | 51,903 | 1.15 (1.11, 1.19) | 1.31 (1.26, 1.36) | 1.23 (1.14, 1.33) |
| Middle | 59,252 | 1.12 (1.09, 1.16) | 1.21 (1.17, 1.25) | 1.24 (1.16, 1.32) |
| Short | 65,164 | 1.13 (1.10, 1.16) | 1.21 (1.18, 1.25) | 1.12 (1.06, 1.19) |
| **TBF (%)** |  |  |  |  |
| Long | 51,710 | 1.17 (1.13, 1.22) | 1.90 (1.82, 1.98) | 1.12 (1.03, 1.23) |
| Middle | 59,024 | 1.10 (1.07, 1.13) | 1.81 (1.74, 1.87) | 1.09 (1.01, 1.17) |
| Short | 64,942 | 1.15 (1.12, 1.18) | 1.71 (1.66, 1.77) | 1.04 (0.98, 1.10) |

Resulted presented as the hazard ratio (with 95% confidence interval). All hazard ratios are adjusted for age, Townsend Deprivation Index, thyroid status, circulatory system disorders, nervous system disorders, smoking status, alcohol use, systolic blood pressure, number of medications taken, blood pressure medication and cholesterol-lowering medication. CAD: 26,127 events; T2D: 19,000 events; IS: 4,842 events.

*Abbreviations:* ABSI, a body shape index; BMI, body mass index; CAD, coronary artery disease; IS, ischemic stroke; TBF, total body fat; T2D, type 2 diabetes; WHR, waist-to-hip ratio.

**Table s10. Associations between different measures of adiposity and cardiometabolic outcome in women, stratified by leukocyte telomere length**

|  |  | **Women** | | |
| --- | --- | --- | --- | --- |
|  | *n* | CAD | T2D | IS |
| **BMI (kg/m^2^)** |  |  |  |  |
| Long | 81,727 | 1.12 (1.08, 1.16) | 1.69 (1.65, 1.74) | 1.05 (0.97, 1.14) |
| Middle | 74,378 | 1.06 (1.03, 1.10) | 1.67 (1.62, 1.72) | 1.02 (0.94, 1.10) |
| Short | 68,466 | 1.07 (1.03, 1.11) | 1.67 (1.62, 1.72) | 1.04 (0.97, 1.12) |
| **ABSI** |  |  |  |  |
| Long | 81,699 | 1.20 (1.15, 1.24) | 1.43 (1.37, 1.48) | 1.17 (1.07, 1.27) |
| Middle | 74,359 | 1.14 (1.10, 1.18) | 1.51 (1.45, 1.57) | 1.15 (1.06, 1.24) |
| Short | 68,456 | 1.16 (1.12, 1.20) | 1.44 (1.39, 1.48) | 1.10 (1.02, 1.19) |
| **WHR adj. BMI** |  |  |  |  |
| Long | 81,727 | 1.13 (1.09, 1.18) | 1.19 (1.15, 1.24) | 1.11 (1.03, 1.21) |
| Middle | 74,378 | 1.13 (1.09, 1.17) | 1.27 (1.22, 1.32) | 1.13 (1.04, 1.22) |
| Short | 68,466 | 1.11 (1.08, 1.15) | 1.28 (1.24, 1.33) | 1.02 (0.95, 1.10) |
| **TBF (%)** |  |  |  |  |
| Long | 81,699 | 1.21 (1.16, 1.26) | 1.91 (1.82, 2.00) | 1.13 (1.03, 1.24) |
| Middle | 74,359 | 1.13 (1.09, 1.18) | 1.87 (1.79, 1.96) | 1.06 (0.98, 1.16) |
| Short | 68,456 | 1.12 (1.07, 1.16) | 1.80 (1.72, 1.88) | 1.10 (1.02, 1.19) |

Resulted presented as the hazard ratio (with 95% confidence interval). All hazard ratios are adjusted for age, Townsend Deprivation Index, thyroid status, circulatory system disorders, nervous system disorders, smoking status, alcohol use, systolic blood pressure, number of medications taken, blood pressure medication and cholesterol-lowering medication. CAD: 26,127 events; T2D: 19,000 events; IS: 4,842 events.

*Abbreviations:* ABSI, a body shape index; BMI, body mass index; CAD, coronary artery disease; IS, ischemic stroke; TBF, total body fat; T2D, type 2 diabetes; WHR, waist-to-hip ratio.

**Table s11. Associations between different measures of adiposity and cardiometabolic outcome in the total study population, stratified by leukocyte telomere length**

|  |  | **Total** | | |
| --- | --- | --- | --- | --- |
|  | *n* | CAD | T2D | IS |
| **BMI (kg/m^2^)** |  |  |  |  |
| Long | 133,630 | 1.10 (1.08, 1.13) | 1.69 (1.66, 1.73) | 1.05 (0.99, 1.11) |
| Middle | 133,630 | 1.07 (1.05, 1.10) | 1.66 (1.63, 1.69) | 1.02 (0.97, 1.08) |
| Short | 133,630 | 1.09 (1.07, 1.12) | 1.62 (1.59, 1.65) | 1.00 (0.96, 1.05) |
| **ABSI** |  |  |  |  |
| Long | 133,409 | 1.20 (1.16, 1.23) | 1.45 (1.40, 1.50) | 1.24 (1.16, 1.33) |
| Middle | 133,383 | 1.16 (1.13, 1.20) | 1.40 (1.36, 1.46) | 1.24 (1.16, 1.32) |
| Short | 133,398 | 1.17 (1.14, 1.20) | 1.39 (1.35, 1.43) | 1.16 (1.09, 1.22) |
| **WHR adj. BMI** |  |  |  |  |
| Long | 133,630 | 1.20 (1.16, 1.25) | 1.35 (1.30, 1.40) | 1.24 (1.14, 1.34) |
| Middle | 133,630 | 1.19 (1.15, 1.22) | 1.35 (1.31, 1.40) | 1.26 (1.17, 1.36) |
| Short | 133,630 | 1.18 (1.15, 1.21) | 1.36 (1.32, 1.41) | 1.11 (1.04, 1.19) |
| **TBF (%)** |  |  |  |  |
| Long | 133,409 | 1.27 (1.23, 1.32) | 2.41 (2.31, 2.51) | 1.18 (1.08, 1.28) |
| Middle | 133,383 | 1.16 (1.13, 1.20) | 2.31 (2.22, 2.41) | 1.11 (1.03, 1.20) |
| Short | 133,398 | 1.20 (1.16, 1.23) | 2.18 (2.10, 2.26) | 1.09 (1.02, 1.17) |

Resulted presented as the hazard ratio (with 95% confidence interval). All hazard ratios are adjusted for age, Townsend Deprivation Index, thyroid status, circulatory system disorders, nervous system disorders, smoking status, alcohol use, systolic blood pressure, number of medications taken, blood pressure medication and cholesterol-lowering medication. CAD: 26,127 events; T2D: 19,000 events; IS: 4,842 events.

*Abbreviations:* ABSI, a body shape index; BMI, body mass index; CAD, coronary artery disease; IS, ischemic stroke; TBF, total body fat; T2D, type 2 diabetes; WHR, waist-to-hip ratio.
