# Supplementary figures and images for: The association of measures of body shape and adiposity with incidence of cardiometabolic disease from an ageing perspective
Source: GeroScience. 2022 Sep 21;45(1):463–76. doi: 10.1007/s11357-022-00654-9 (PMC9886769; doi:10.1007/s11357-022-00654-9)

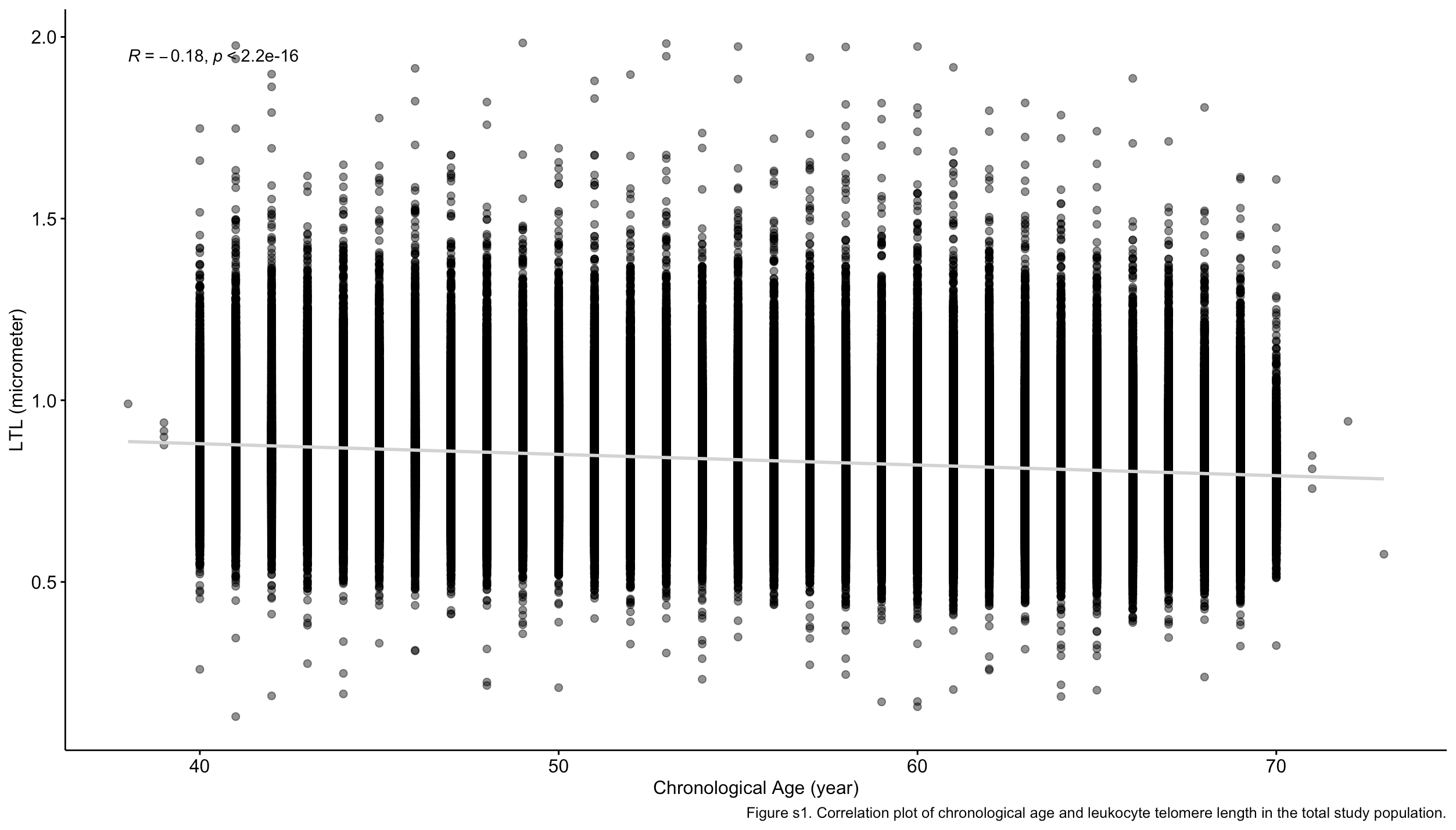

Supplement: Supplementary file 2 — (PNG 328 kb) [file 11357_2022_654_Fig4_ESM.png]

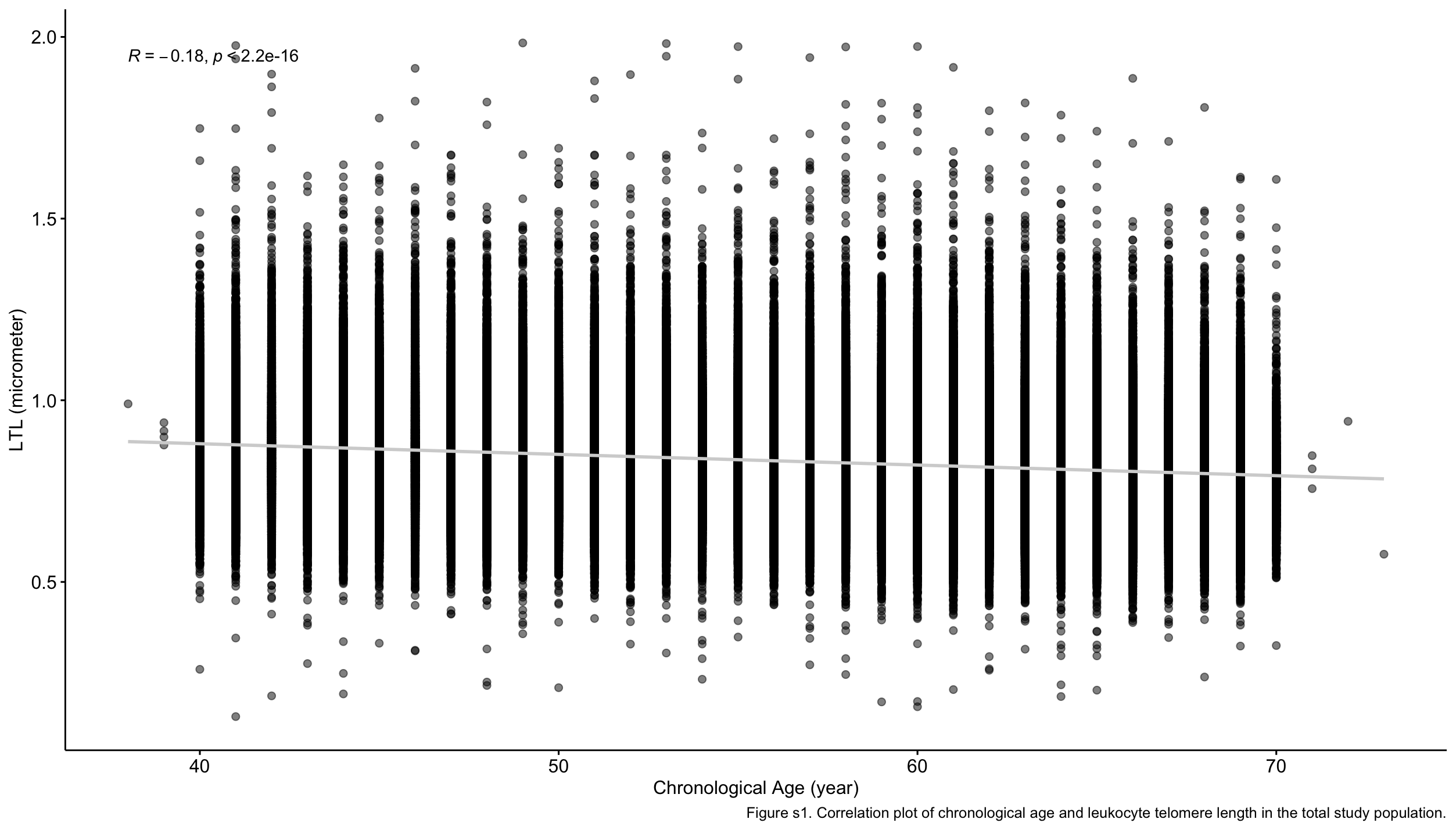

Supplement: Supplementary file 3 — High resolution image (TIFF 401 kb) [file 11357_2022_654_MOESM2_ESM.tiff]
